# Structural and mechanistic basis of mTORC2 activation of protein kinase AKT/PKB

**DOI:** 10.1042/BCJ20253108

**Published:** 2026-03-03

**Authors:** Nam Chu, Nhat Le, Ouada Nebie, Sammi Yang

**Affiliations:** 1 Department of Cancer Biology and Genetics, College of Medicine, The Ohio State University, Columbus, OH 43210, U.S.A.; 2 Comprehensive Cancer Center, The Ohio State University Wexner Medical Center, Columbus, OH 43210, U.S.A.

**Keywords:** AKT, cell signaling, mTOR, posttranslational modifications, protein kinase

## Abstract

The PI3K/AKT/mTOR signaling pathway is crucial for regulating essential cellular processes such as growth, survival, metabolism, and protein synthesis. Dysregulation of this pathway is strongly associated with diseases like cancer, where it drives uncontrolled cell proliferation and survival. The mTOR kinase forms two multiprotein complexes, mTORC1 and mTORC2, which govern distinct signaling pathways. mTORC1, regulated by nutrients, controls protein synthesis, cell growth, and autophagy, while mTORC2 acts as a central node in phosphoinositide 3-kinase (PI3K) and Ras signaling, often disrupted in cancer and diabetes. AKT, recruited by PIP3 to the plasma membrane, is phosphorylated by PDK1 and mTORC2, enabling it to regulate various cellular functions. Notably, mTORC2 selectively phosphorylates AKT and PKC but no other closely related kinases targeted by mTORC1, reflecting a high degree of substrate specificity. This specificity is due to structural elements in AKT that interact with the mTORC2 subunit mSin1 as revealed by recent studies using semisynthetic probes, paving the way for the design of mTORC2-specific inhibitors. Given the pathway’s significant role in disease progression, particularly cancer, targeting the AKT/mTOR axis holds considerable therapeutic promise. However, challenges remain due to the complex regulation and feedback mechanisms in this pathway. Emerging combination therapies show promise in overcoming these obstacles. This review highlights the intricate regulation of the AKT/mTOR pathway and its potential for developing targeted therapies.

## Introduction

The PI3K/AKT/mTOR signaling pathway plays a central role in regulating key cellular processes such as growth, survival, metabolism, and protein synthesis. Dysregulation of this pathway is closely linked to various diseases, particularly cancer, where it drives uncontrolled cell proliferation and survival. In this review, we provide a comprehensive overview of the molecular mechanisms governing AKT activation, its regulation by phosphoinositide 3-kinase (PI3K), and the roles of the mTOR complexes in cellular homeostasis. AKT, a crucial downstream effector of PI3K, is activated by the accumulation of PIP3 at the plasma membrane, which facilitates its recruitment and phosphorylation by 3-phosphoinositide-dependent protein kinase-1 (PDK1) and mTORC2, leading to its full activation.

We also explore the complex interplay between AKT and the mTOR complexes, mTORC1 and mTORC2. mTORC1, which regulates protein synthesis, cell growth and autophagy, is activated by AKT through the inhibition of its negative regulators TSC2 (Tuberous Sclerosis Complex 2) and PRAS40 (Proline-rich Akt substrate of 40 kDa). mTORC2, on the other hand, directly phosphorylates AKT at Ser473, enabling its full activation and subsequent regulation of numerous cellular processes. The PH domain of AKT is crucial for its membrane targeting and activation, playing a role in both its functional activation and autoinhibition.

Additionally, we discuss the therapeutic potential of targeting the AKT/mTOR signaling axis. Given its significant role in disease progression, particularly in cancer, this pathway has become a major target for therapeutic strategies. However, challenges persist in selectively modulating this pathway due to its complex regulation and feedback mechanisms. Emerging therapeutic approaches, including combination therapies, offer promising strategies to overcome these challenges and improve treatment outcomes. By providing insights into the intricate regulation of AKT and its interaction with mTOR complexes, this review aims to inform the development of more effective therapies targeting this critical signaling pathway.

## AKT: a dominant downstream effector of PI3K signaling

### AKT within the AGC kinase family

Protein phosphorylation is one of the most common posttranslational modifications (PTMs) that has been identified in the human proteome and can impact the flow of cellular signaling information from the extracellular space to the nucleus. Among serine and threonine kinases, the AGC kinase family, named after protein kinase A, G, and C, comprises approximately 60 members that regulate cellular growth, metabolism, and survival in response to extracellular stimuli [1,2].

Protein kinases of the AGC family share common structural features within their catalytic kinase domains with the rest of the protein kinase superfamily. The conserved kinase domain contains a small N-terminal lobe possessing a 5-stranded β-sheet and a helix, αC, and a large mostly α-helical C-terminal lobe. Some AGC kinases also possess a second N-lobe helix, αB adjacent to the αC helix. The cleft interface of the two lobes is filled by Mg-ATP and a portion of peptide/protein substrate [1]. Related to peptide/protein substrate recognition, most AGC members are basophilic protein kinases that prefer peptide substrates containing basic amino acid residues (Arg/Lys) N-terminal to the phospho site (Ser/Thr). These positively charged residues make strong interactions with Asp and Glu residues in the small and large lobes of the kinase domain, thus upon binding, the peptide/protein substrate can stabilize the active-closed conformation and release the inhibitory domains in many AGC kinases.

**Figure 1:**
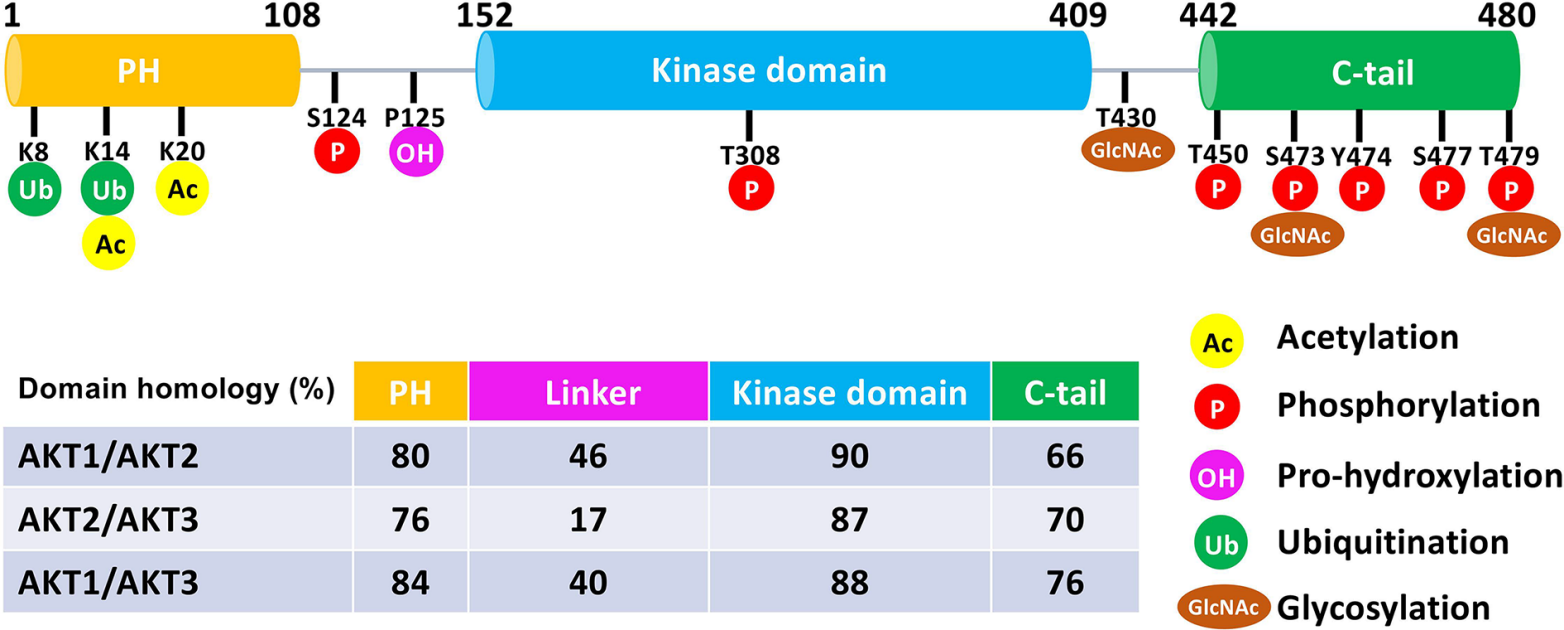
Schematic illustration of AKT PTMs discussed in the current review Domain structure and PTM sites of AKT1 are shown. PH (Pleckstrin homology domain), KD (Kinase domain), and C-tail (regulatory domain). Comparison of Akt domain homology for three AKT isoforms. Domain structure and PTM sites of AKT1 are shown (residue numbering corresponds to the AKT1 isoform).

AKT is a prominent member of the AGC kinase family and functions as a central signaling hub downstream of PI3K. Originally identified as the cellular homolog of the acute transforming retrovirus AKT8 [3–5], AKT regulates cell proliferation, survival, and metabolism in response to the insulin and growth factor signaling pathways. In mammals, the AKT subfamily has three highly conserved but functionally distinct isoforms AKT1, AKT2, and AKT3 [3,6–8] that serve as key nodes in of cell signaling networks. AKT dysregulation is linked to multiple diseases including cancers, metabolic syndrome, neurodegenerative disorders, and immune dysfunction-associated conditions. AKT1 is commonly expressed in the vast majority of tissues, AKT2 has been predominantly found in insulin-sensitive tissues, and the expression of AKT3 has mostly been described in neurons and testes [7]. Each AKT isoform contains an N-terminal PH domain followed by a central kinase domain and the C-terminal regulatory domain, here named the C-tail. Although AKT1-3 display high overall sequence similarity, divergence within the PH-kinase linker and C-terminal tail regions contributes to isoform-specific regulation and substrate selection [9] (Figure 1). Since AKT1-3 have a similar activation mechanism controlled by PI3K, PDK1, and mTORC2, and share some common downstream substrates, it has been proposed that these isoforms might exhibit highly overlapping biological functions, especially for AKT1 and AKT2. Despite their high sequence similarity, the AKT isoforms are involved in distinct physiological and pathological activities, possibly, due to their expression patterns and subtle structural differences. Some evidence suggests that the different AKT isoforms have distinct substrate specificity and specific biological functions [10]. AKT1 is widely expressed and is primarily associated with cell proliferation and survival. AKT2 is the predominant isoform in insulin-responsive tissues and a critical regulator of glucose metabolism. AKT3 is mostly found in the brain and testes and plays an important function in brain development. In breast cancer, for instance, the hyperactivation of AKT1 is suggested to initiate the tumorigenesis, whereas AKT2 appears to be more responsible for tumor progression and metastasis [11].

Thus far, over 100 AKT downstream substrates have been reported but not all of these have been fully established. AKT substrates often contain a consensus motif R-X-R-X-X-S/T-ϕ, where X is any amino acid and ϕ stands for a large hydrophobic amino acid [12]. AKT may up- or down-regulate the functions of these substrates (many of them are often inhibited by AKT), and/or change their subcellular localization or stability [13]. Some well-established AKT substrates include glycogen synthase kinase 3 (GSK3), Forkhead Box O (FOXO) family transcription factors, and TSC2 [12].

### Activation and regulation of AKT by PI3K and PTEN

**Figure 2:**
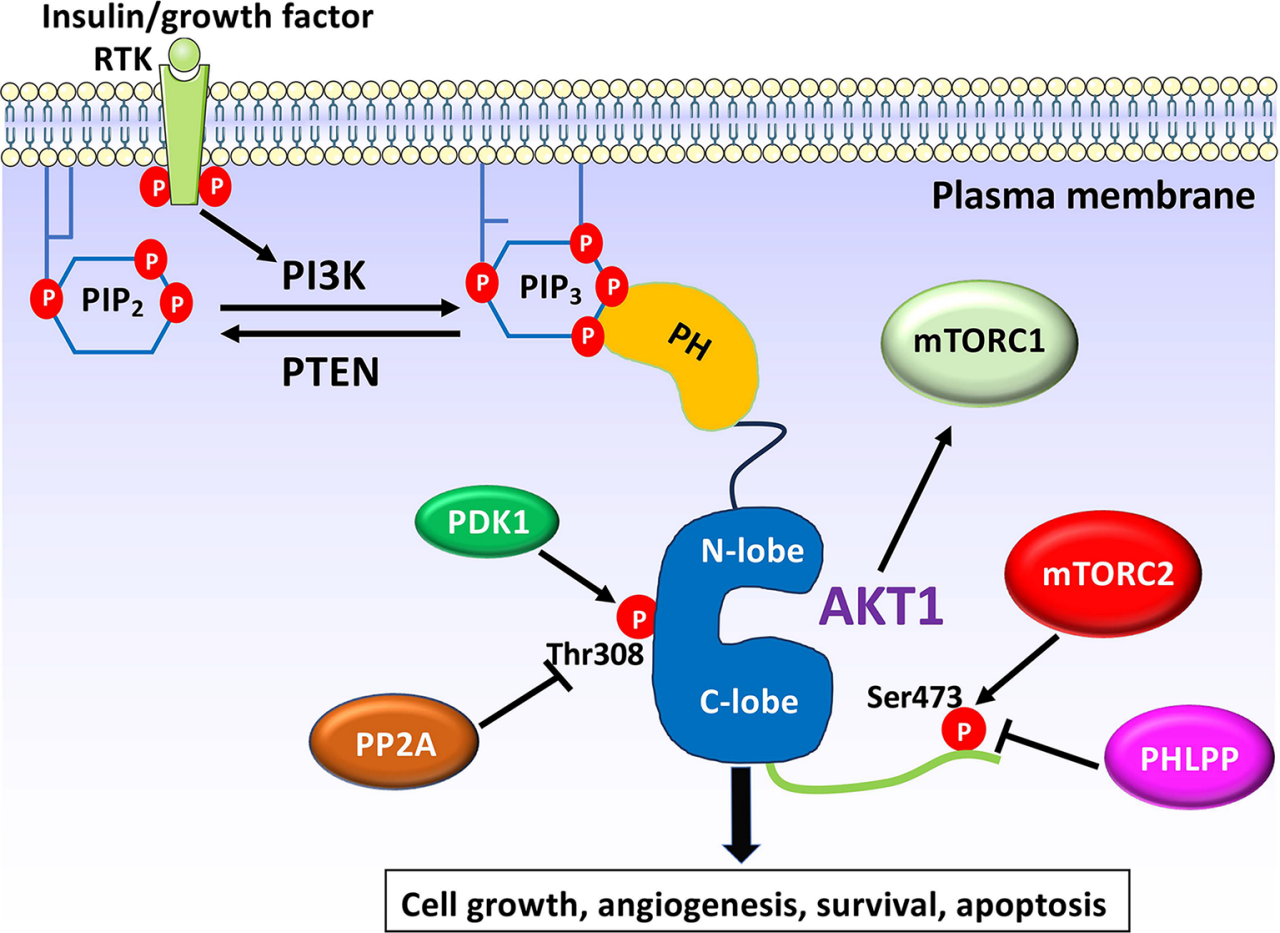
AKT activation via PI3K signaling pathway Stimulation of RTK by insulin or growth factors leads to activation of PI3K, that can generate PIP3 from PIP2 at the plasma membrane. Next, PIP3 recruits inactive AKT form from cytoplasm to membrane via the binding to AKT PH domain. Once at the plasma membrane, AKT is subjected to a series of phosphorylations at Thr308 and Ser473 by PDK1 and mTORC2 complex, respectively, resulting in full activation. This activation signal is terminated by PIP3 phosphatase PTEN and protein phosphatases PP2A and PHLPP.

AKT is specifically activated through a tightly coordinated signaling cascade driven by PI3K [14]. Upon the binding of growth factors to cell surface receptors, such as receptor tyrosine kinases (RTKs), PI3K is activated and catalyzes the conversion of PIP2 (phosphatidylinositol-4,5-bisphosphate) to PIP3 (phosphatidylinositol-3,4,5-trisphosphate) at the plasma membrane. The accumulation of PIP3 at the membrane provides a docking site for proteins that possess PH domains, including AKT and PDK1. Once recruited to the membrane, PDK1 phosphorylates AKT at Thr308 in the activation loop, leading to partial activation of AKT. However, full activation of AKT requires additional phosphorylation at Ser473, located in the C-terminal hydrophobic region of AKT, which is primarily carried out by mTORC2. After phosphorylation at both Thr308 and Ser473, AKT is fully activated and can phosphorylate a variety of downstream targets involved in processes such as cell survival, growth, metabolism, and protein synthesis (Figure 2). It is important to note that AKT’s substrate specificity is strongly influenced by the phosphorylation levels at Ser473 and Thr308 [15,16,17]. The ratio of phosphorylation at these two sites determines which substrates AKT preferentially targets [12,18–20]. While phosphorylation at Thr308 is critical for kinase activity and serves as the primary determinant for substrate recruitment, phosphorylation at Ser473 helps fine-tune substrate preference. AKT with both phosphorylation sites exhibits a broader substrate selection than either site is phosphorylated alone [21].

In addition to PIP3, another PI3K product, PI3,4P2, has also been identified as an important regulator of AKT. PI3,4P2 is typically produced from PIP3 by lipid phosphatases, including SHIP (SH2-containing inositol phosphatase) and INPP5 (inositol polyphosphate-5-phosphatase) [22,23] or synthesized directly by class II PI3Ks [24]. Notably, PI3,4P2 specifically regulates the recruitment of AKT2 to the membrane [25], while PIP3 exclusively recruits AKT1 and AKT3, suggesting isoform-specific signaling [26]. Recent studies have shown that the linker region between AKT’s PH and kinase domains plays a key role in determining the specificity for PIP3 and PI3,4P2 binding, with AKT1 and AKT3 preferentially interacting with PIP3 and AKT2 with PI3,4P2 [26,14]. The functional significance of PI3,4P2 in AKT activation, particularly in isoform-specific regulation, remains an ongoing area of research.

PTEN (phosphatase and tensin homolog) serves as a critical negative regulator of PI3K signaling. PTEN dephosphorylates PIP3 back to PI4,5P2, thereby inhibiting AKT recruitment to the plasma membrane and preventing its activation (Figure 2). PTEN activity is regulated at both the transcriptional and post-translational levels. NF-kB (nuclear factor kappa-light-chain-enhancer of activated B cells) regulates PTEN transcription [27,28], while NEDD4-1 (an E3 ubiquitin ligase) targets PTEN for proteasomal degradation [29–31]. In addition, PTEN function is modulated by PTMs, including phosphorylation of its C-terminal tail, which influences PTEN stability, subcellular localization, and lipid phosphatase activity [32,33]. Mutations or dysregulation of PTEN lead to elevated levels of PIP3, which results in the hyperactivation of AKT, contributing to tumor cell proliferation. PTEN loss is common in many human cancers, and its dysfunction represents an important target for therapeutic intervention [34,35].

### Spatial control of AKT signaling outputs

Initially, it was thought that AKT activation is heavily reliant on its recruitment to the plasma membrane by PIP3 and PI3,4P2, which are essential for its full activation [32, 36–40]. However, subsequent studies identified AKT activation extends to multiple cellular compartments, including the cytosol, mitochondria, lysosomes, and the nucleus [12,41–45]. These findings raised questions regarding the spatial requirements for AKT activity beyond its traditional role at the plasma membrane. Importantly, additional studies have shown that sustained AKT activity is largely confined to the membrane-associated compartments, upon detachment from membranes, AKT is rapidly dephosphorylated by phosphatases such as PHLPP (phosphatase and tensin homolog-induced putative phosphatase) and PP2A [46,47], rendering it inactive. This coupling of phosphorylation to membrane localization ensures tight spatial control of AKT activity. Nevertheless, AKT can exert regulatory effects at membrane-distal locations. Some earlier studies have identified nuclear functions of AKT [48], particularly in the regulation of transcriptional factors like FOXO family members and the coactivator p300 [49–54]. For instance, phosphorylation of FOXO1A by AKT in the nucleus results in its export from the nucleus to the cytoplasm, thereby inhibiting its transcriptional activation of gluconeogenesis [49,54,55]. These observations indicate that while AKT activation is spatially restricted to membrane compartments, its downstream regulatory functions can extend to membrane-distal cellular locations, including the nucleus.

In addition to the interaction of AKT with PIP3 and PI3,4P2, phosphatidylserine (PS) has been recognized as a crucial modulator of AKT activation at the membrane [56–58]. PS binds specific residues in the PH domain and C-tail of AKT, promoting the binding of AKT to PIP3 and facilitating its activation [56,57] (Figure 2). Disruption of this interaction, such as through mutagenesis of Arg15 and Lys20 within the PH domain, impairs AKT activation and subsequent cell survival, particularly under conditions with limited PIP3 availability [56].

## mTOR complexes and AKT: structural and functional integration

### mTORC1 and mTORC2: architecture, localization, and function

mTOR is a conserved serine/threonine kinase belonging to the phosphoinositide 3-kinase-related kinase (PIKK) family and functions as a central regulator of cell growth, metabolism, protein synthesis, autophagy, and survival [40,59–61]. mTOR exerts its functions through two distinct, yet interconnected, multiprotein complexes: mTORC1 and mTORC2, which are physically and functionally distinct but share mTOR as a catalytic subunit [61], and two other core subunits: mLST8 (mammalian lethal with Sec13 protein 8) and Deptor (dep domain-containing mTOR-interacting protein) [62,63]. In addition to these common subunits, mTORC1 and mTORC2 contain distinct complex-specific proteins. PRAS40 and Raptor (mTOR regulatory-associated protein) are mTORC1-specific subunits [64–68] whereas mSin1 (mitogen-activated protein kinase-associated protein 1), Rictor (rapamycin-insensitive companion of mTOR), and Protor (protein observed with Rictor) are mTORC2 unique subunits [61,69–71].

mTORC1 localizes predominantly to the cytoplasm and is enriched on lysosomal membranes, where it integrates inputs from nutrient availability, cellular energy status, and stress. It can also be found in the cytosol and at the endoplasmic reticulum (ER). Furthermore, the activation of the mTORC1 pathway by amino acids is associated with the movement of mTORC1 from an undefined location to a compartment containing Rab7, which is a marker for both late endosomes and lysosomes [72,73]. mTORC1 activation plays a central role in regulating protein synthesis, cell growth, lipid metabolism, and autophagy [59,61,74]. In contrast, mTORC2 localizes to multiple intracellular compartments, including the plasma membrane, ER, mitochondria-associated ER membranes, endosomes, lysosomes, nucleus, and Golgi apparatus [75–77]. mTORC2 is activated downstream of growth factor and PI3K signaling and primarily regulates cell survival, metabolism, and cytoskeletal organization through phosphorylation of AGC family kinases, including AKT, SGK, and PKC [12,60,61,74].

### AKT activation of mTORC1

**Figure 3:**
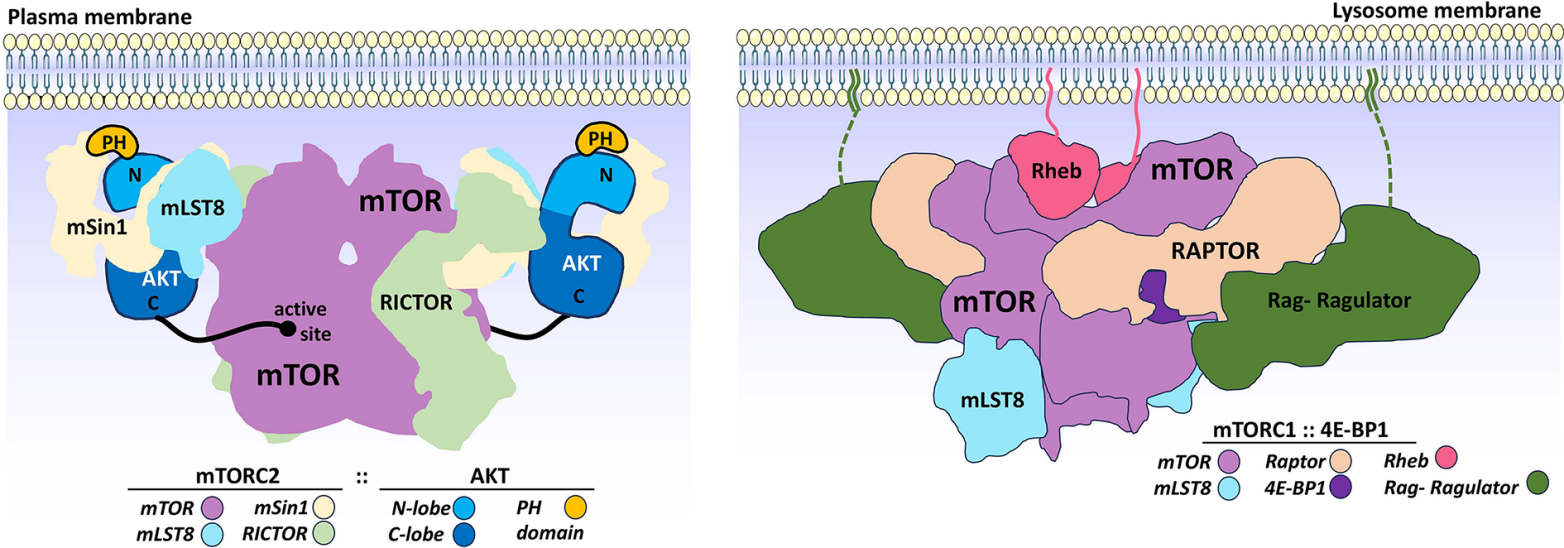
A schematic illustration shows substrate-bound mTORC2 at the plasma membrane and mTORC1 at the surface of the lysosome Both mTORC2 mSin1’s and AKT’s PH domains interact directly with the plasma membrane via PIP3. In contrast, several lipid anchors on the small G-protein Rheb and the Rag–Ragulator complex tether mTORC1 to the lysosomal membrane. Both the Rag–Ragulator complex and Rheb are associated with the mTORC1-specific component Raptor, which also recognizes the substrate 4E–BP1. mTOR and mLST8 are shared components between the two complexes (adapted from M. S. Taylor et al., 2025).

AKT serves as a key upstream regulator of mTORC1 and links growth factor signaling to anabolic metabolism and cell growth. One principal mechanism by which AKT activates mTORC1 is through phosphorylation and inhibition of the TSC1–TSC2 complex [78–81]. In its inactive state, TSC2, when bound to TSC1, acts as a GTPase-activating protein (GAP) for Rheb (Ras homolog enriched in brain), inhibiting the activation of Rheb–GTP. Rheb–GTP is essential for activating mTORC1. AKT phosphorylates and inactivates TSC2, which leads to the activation of Rheb–GTP, consequently activating mTORC1. AKT also directly phosphorylates PRAS40, a negative regulator of mTORC1, promoting its dissociation from the complex and further enhancing mTORC1 activity [67,82,83]. Once activated, mTORC1 phosphorylates canonical substrates such as S6K1 and 4E–BP1, leading to enhanced protein synthesis, ribosome biogenesis, and cell growth through regulation of translation initiation and elongation [84–86]. The Raptor subunit of mTORC1 plays an essential role in substrate recognition. Raptor is responsible for recruiting substrates to the mTORC1 complex by recognizing specific linear sequence motifs present in canonical mTORC1 substrates like S6K1 and 4E–BP1 [87–92]. The recruitment of these substrates to mTORC1 facilitates their phosphorylation and activation, promoting essential processes such as translation initiation, ribosome biogenesis, and cell growth (Figure 3, at lysosome). It is critical to note that, phosphorylation of TSC2 and PRAS40 by AKT can occur when AKT is phosphorylated at Thr308 alone, although Ser473 phosphorylation enhances catalytic efficiency and broadens substrate engagement. This feature contributes to the complexity of AKT-mediated regulation of mTORC1 under different signaling contexts.

### Structural basis of AKT recognition and phosphorylation by mTORC2

In addition to acting upstream of mTORC1, AKT is itself a direct substrate of mTORC2 (Figure 3, at cytoplasm). This complex phosphorylates AKT at Ser473, an essential step for AKT to become fully activated and maximal signaling output [40,93–95]. Although one study proposed that Ser473 autophosphorylation arises indirectly following mTORC2-dependent phosphorylation of a priming site in the ‘TIM’ motif (Thr443 in AKT) [96], recent biochemical analyses using purified mTORC2 and homogeneous AKT Ser473 substrate demonstrate that mTORC2 directly catalyzes phosphorylation of Ser473 [97].

**Figure 4:**
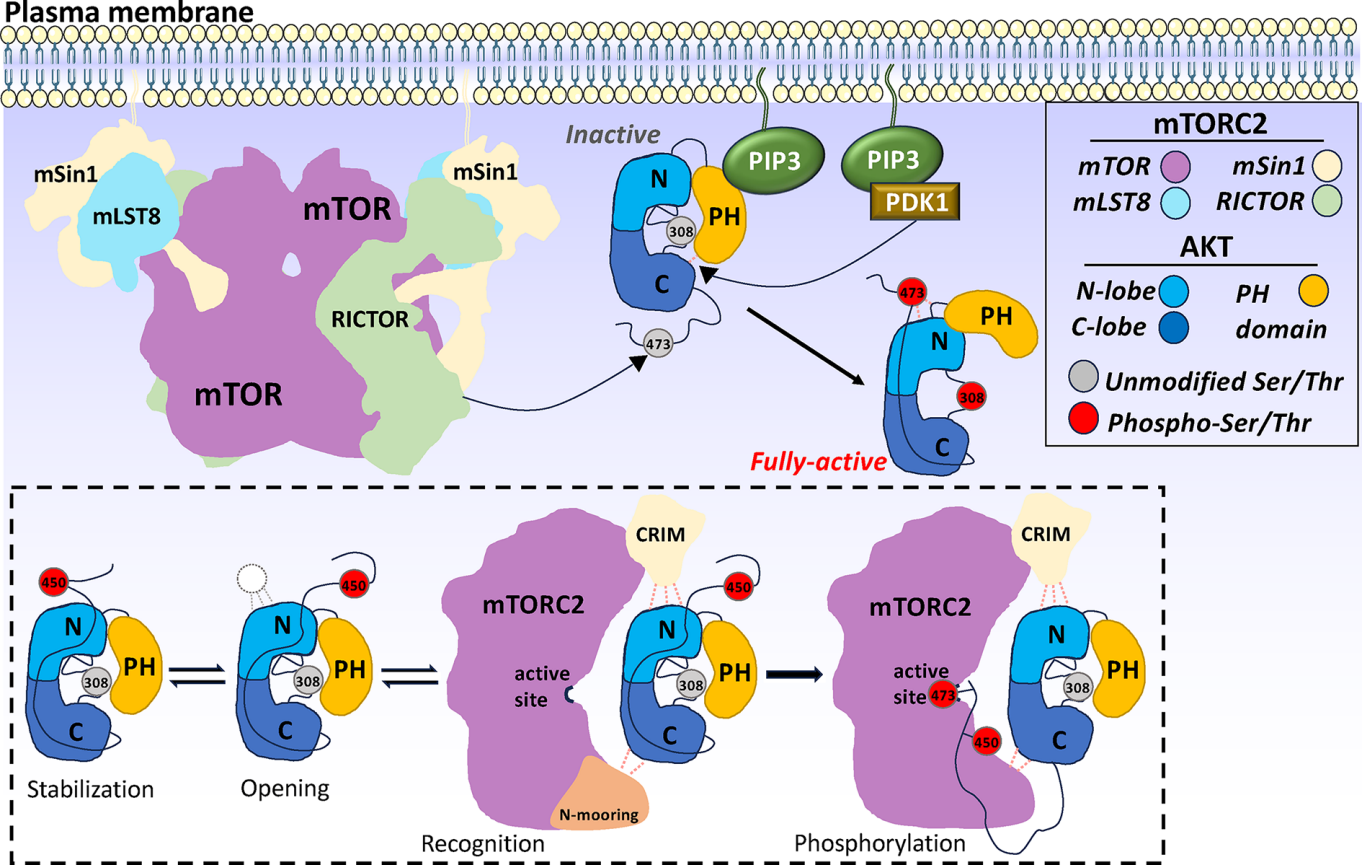
A conceptual model for mTORC2 activation of AKT at the plasma membrane, with mTORC2 specifically phosphorylating Ser473 of AKT Upon PIP3-mediated recruitment, AKT is subjected to a series of modifications: (1) mTORC2 phosphorylates Ser473 in the hydrophobic motif, causing a conformational shift in the PH domain and (2) PDK1 phosphorylates Thr308 in the activation loop, leading to AKT full activation. The dashed box illustrates that AKT kinase domain N-lobe engages regulatory subunits within mTORC2 complex, including mSin1, in a dynamic manner influenced by phosphorylation at Thr450, which stabilizes AKT while modulating accessibility of this interaction surface. Sufficient docking of the AKT kinase domain enables positioning of Ser473 into the mTOR active site for phosphorylation, integrating membrane localization, conformational dynamics, and multisite phosphorylation to achieve robust AKT activation (adapted from M. S. Taylor et al., 2025).

In the recognition of AKT substrate by mTORC2, the regulatory subunit mSin1 plays a critical role. This recognition is mediated through two distinct long-range docking interfaces: the mSin1 CRIM (conserved region in the middle) interacting with the N-lobe of AKT kinase domain (KD), and the mSin1 N-terminal region (N-mooring) interacting with AKT KD C-lobe. These interfaces are distal to the mTOR active site (Figure 4) [63,97]. The mSin1 CRIM domain allows mTORC2 to phosphorylate substrates at hydrophobic motifs, such as Ser473 in AKT and Ser338 in SGK [98–100]. Additionally, mTORC2 stabilizes certain phosphorylation events, such as the turn motif phosphorylation on AKT (Thr450) [98–100], which stabilizes the active conformation of AKT, enabling it to perform its cellular functions effectively.

This docking-based recognition mechanism allows mTORC2 to phosphorylate substrates with diverse primary sequences and explains its selectivity for AGC kinases such as AKT, SGK, and PKC [12,40,101–103].

### Feedback regulation, substrate specificity, and cross-talk of AKT and mTOR complexes

The AKT–mTOR signaling network is regulated by multiple feedback loops that constrain signaling amplitude and duration, AKT activation of mTORC1 and its downstream effector S6K1 leads to phosphorylation of insulin receptor substrates including IRS1 and IRS2, the major activators of PI3K signaling. This phosphorylation event negatively regulates IRS-1 and IRS-2 thereby limiting PI3K activity and preventing excessive mTORC1 activation [104–112]. This negative feedback loop ensures that mTORC1 activity is tightly controlled and prevents uncontrolled cell growth and proliferation. In parallel, mTORC2 has been implicated in feedback regulation of PI3K and RTKs. This regulation ensures that the entire AKT–mTOR signaling axis remains balanced, helping maintain cellular homeostasis [113,114].

Substrate recognition strategies differ fundamentally between mTORC1 and mTORC2 [59–61]. mTORC1 recognizes a defined linear motif in its substrates called the TOR signaling (TOS) motif. This motif contains hydrophobic and basic residues, and the complex typically requires a priming phosphorylation event to be detected and efficiently phosphorylated by the complex. In contrast, mTORC2 employs three dimensional docking interactions mediated by mSin1 to engage its substrates such as AKT and other AGC kinases (Figure 4). Local sequence plays little role in this process, consistent with mTORC2 phosphorylation of adjacent AKT C-tail residues (Ser475, Ser477, and Thr479) at lower rates [115]. Notably, the same basic surface on AKT that binds intramolecularly to phosphorylated Thr450, thereby stabilizing the kinase and protecting it from dephosphorylation, also mediates binding to mTORC2 [97,98,116] (Figure 4). This dual functionality provides a mechanistic explanation for how mTORC2 recognizes substrates with limited sequence conservation.

Together, these mechanisms illustrate how AKT functions as a central integrator of mTORC1 and mTORC2 signaling, coordinating growth factor inputs with metabolic and survival responses while maintaining tight regulatory control.

## Functions of PH domain in mTORC2 activation of AKT

### General properties of PH domains

PH domains are small protein modules, typically around 120 amino acids, that mediate phospholipid-protein interactions in a variety of proteins involved in intracellular signaling pathways. PH domains were initially discovered as an internal repeat in the pleckstrin domain, a major substrate of PKC in platelets [117–119]. Subsequent studies revealed the presence of this domain in many other proteins with diverse roles in cell signaling and cytoskeleton organization [120,121]. Despite significant divergence in amino acid sequence, PH domains share a conserved tertiary structure consisting of a 7-stranded β-sandwich followed by a C-terminal amphipathic helix [122]. The C-terminal helix closes one side of the β-sandwich structure. The close proximity of the N- and C-terminal residues enables the PH domain to function as a relatively independent module, embedded within larger proteins and connected to other protein domains by dynamic linkers [123].

Although PH domains are commonly associated with phosphoinositide binding, only a subset display high affinity and specificity for membrane lipids. These lipid-binding PH domains preferentially recognize phosphoinositides containing adjacent phosphate groups within the inositol headgroup, including PI4,5P2, PI3,4P2, and PI3,4,5P3 [124]. Structural studies have revealed that phosphoinositide recognition is mediated by clusters of basic residues that engage phosphate groups through extensive hydrogen bonding networks [125–127].

### PH domain-mediated membrane targeting of AKT

A well-characterized example of PH domain function is its role in the localization of AKT, a serine/threonine kinase, to the plasma membrane (Figure 2). Despite this, the exact process of AKT’s translocation from the cytosol for subsequent activation by PDK1 and mTORC2 is not yet fully understood

Early models proposed that PH domain of inactive Akt interacts with cytoplasmic protein adaptors, facilitating its recruitment to the plasma membrane [128,129]. More recently, ubiquitin-like protein 4A (UBL4A) was identified as a factor required for efficient AKT membrane localization and activation following insulin stimulation [130]. In this study, UBL4A promotes actin branching through interaction with the Arp2/3 complex, thereby positioning AKT in proximity to the plasma membrane. Consistent with this model, the AKT PH domain has been shown to bind filamentous actin and colocalize with Arp2/3 at membrane-associated actin structures [130].

### PH domain-driven autoinhibition of AKT

In addition to membrane targeting, the AKT PH domain also regulates autoinhibition of AKT’s kinase activity. The PH domain interacts intramolecularly with the kinase domain, maintaining the kinase in an inactive state [2,131]. This autoinhibition model was supported by a 2.7 Å resolution co-crystal structure of C-terminally truncated AKT1 (aa 1–443), containing both PH and kinase domains in complex with allosteric inhibitor VIII [131]. This structure revealed a stable PH-kinase interface that constrains the kinase in an inactive state (Figure 4).

The binding of PIP3 to the PH domain of full-length AKT has been hypothesized to release AKT from its autoinhibited state by dislodging the PH domain from the kinase domain. This hypothesis was partially confirmed by biochemical studies using purified AKT and PIP3-containing vesicles [47], although direct activation of AKT by PIP3 could not be replicated in lipid vesicle kinase assays [2,103]. These observations suggest that additional factors, including membrane context and higher-order interactions, may contribute to PH domain-mediated regulation.

### Structural and biochemical models of AKT PH-kinase regulation

The structure of VIII-bound AKT1 has been used as a model for the natural autoinhibited form of AKT. The binding of compound VIII seems to drive the C-lobe of the kinase domain, disrupting the phospholipid-binding site of the PH domain. In contrast, inactive Akt demonstrated a potent binding affinity for soluble, fluorescein-labeled PIP3 [103]. To address this discrepancy, a segmental isotopic labeling approach was employed to analyze AKT1 by NMR [132]. The NMR data revealed that the PH domain adopts distinct conformations in inactive versus inhibitor-bound AKT, as evidenced by significant chemical shift perturbations in the PH domain of each AKT phospho-forms. Fluorescence anisotropy binding assays using fluorescein-labeled PIP3 in the presence or absence of the AKT allosteric inhibitor MK2206 (similar action as compound VIII) showed that MK2206 reduced the binding affinity of PIP3 to AKT by more than 30-fold. This suggests that MK2206 may affect the PH domain conformation and prevent AKT from reaching the plasma membrane, thereby inhibiting cellular activation [132]. Recent research has identified a network of PH domain residues, including Arg86, Glu17, and Tyr18, which are critical in regulating autoinhibition [133]. This regulation may be mediated, in part, through a Tyr18/Phe309 aromatic interaction [134]. In parallel, a separate study reported a crystal structure of engineered human AKT1 stabilized by a nanobody, yielding valuable structural insight into model of PH-driven autoinhibition [46,135]. Using this engineered AKT1 construct, the study demonstrated that the PH domain forms a stable intramolecular interface with the kinase domain that persists despite activation loop and hydrophobic motif phosphorylation, thereby maintaining an autoinhibited conformation in the absence of phosphoinositide binding. These findings support a model in which lipid engagement is required to relieve PH-mediated autoinhibition and highlight the importance of intramolecular domain interactions in constraining AKT activity.

### mTORC2 and AKT docking at the plasma membrane

The process of AKT translocation and activation is also influenced by mTORC2, a multi-protein complex involved in regulating AKT. The docking of mTORC2 and AKT to the plasma membrane depends on the presence of four PH domains within the mTORC2 complex. Interestingly, the orientation of mTOR in mTORC2 at the plasma membrane is the opposite of mTORC1’s orientation at the lysosome surface [65,89,91]. The orientation of mTORC1 is anchored by Rheb binding mTOR and Rag GTPases binding to Raptor (Figure 3). However, Raptor is absent in mTORC2, and Rheb cannot interact with mTORC2 [136,137]. These unique orientations could contribute to the *in vivo* specificities of mTOR complexes [138]. While these data were obtained in solution, not at the membrane, more research is required to understand mTORC2 regulation in physiology and how its dysregulation contributes to diseases such as diabetes and cancers[139–141].

## Implications for cancer and therapeutic targeting

The AKT/mTOR signaling pathway plays a central role in cancer development and progression, making it a critical target for therapeutic intervention. Dysregulation of this pathway, often through mutations in key regulatory proteins or loss of tumor suppressors, results in uncontrolled cell growth and survival characteristic of cancer. Specifically, PI3K hyperactivation and the loss-of-function mutations in PTEN are frequently observed in many cancers, including breast, prostate, glioblastoma, and endometrial cancers. PTEN normally inhibits PI3K signaling, and loss of its function through mutations, epigenetic silencing, or PTMs lead to the constitutive activation of AKT isoforms, which contributes to tumorigenesis. As a result, the AKT/mTOR pathway has become a promising target for therapeutic strategies aimed at disrupting this dysregulated signaling to halt cancer progression.

### Current therapeutic strategies targeting the AKT/mTOR pathway

Several therapeutic strategies are currently being explored to target the AKT/mTOR pathway in cancer. PI3K inhibitors, such as idelalisib, copanlisib, and duvelisib, have been FDA-approved, primarily for treating follicular lymphoma [142]. These inhibitors act upstream of AKT, blocking the activation of the pathway and helping to reduce tumor growth driven by PI3K hyperactivation. However, while promising, the use of these inhibitors is limited to cancers where PI3K activation is a primary driver. In addition to PI3K inhibitors, AKT inhibitors have been developed to directly target and block the activity of AKT isoforms. Although the therapeutic targeting of AKT has historically faced significant challenges, recent clinical progress has led to the FDA approval of the ATP-competitive AKT inhibitor capivasertib (AZD5363) in combination regimens for selected cancer indications [143]. In parallel, multiple pan-AKT inhibitors continue to be evaluated in clinical trials, including ATP-competitive compounds such as GSK2141795 and GDC0068, as well as allosteric inhibitors including ARQ092, BAY1125976, MK-2206, and TAS-117.

### mTOR inhibitors and pathway feedback

In addition to AKT inhibitors, mTOR inhibitors such as rapamycin and its derivatives, known as rapalogs, are commonly used to target mTORC1 through FKBP12-dependent binding [144] and generally exert minimal direct effects on mTORC2, as steric constraints limit access of the FKBP12–rapamycin complex to the mTOR FRB domain within mTORC2. On the other hand, ATP-competitive inhibitors such as Torin 1 [145] and Torin 2 [146] target the mTOR catalytic site and inhibit both mTOR complexes due to the nearly identical configuration of the active site in the two complexes. Although this broader inhibition can more effectively suppress oncogenic signaling, concomitant inhibition of mTORC2 disrupts AKT signaling contributing to insulin resistance and diabetes-like phenotypes [147]. These feedback mechanisms underscore the challenge of achieving effective and selective therapeutic modulation of the mTOR pathway. As a result, there is growing interest in developing selective inhibitors that specifically target mTORC2. This would allow for more precise treatments, minimizing the side effects associated with broad inhibition of the mTOR pathway.

### Future directions: selective targeting and combination therapies

Looking ahead, the development of selective mTORC2 inhibitors offers an exciting direction for cancer therapy. By targeting mTORC2’s unique features, such as its interaction with AKT through the mSin1 CRIM domain (Figure 4), one hopes to develop inhibitors that specifically block mTORC2 activity without affecting mTORC1. This would provide more precise and effective treatment for cancers driven by mTORC2 dysregulation, reducing the risk of side effects that are common with broad mTOR inhibitors. In parallel, combining AKT/mTOR inhibitors with immunotherapy or chemotherapy, could address multiple pathways involved in cancer progression, leading to better patient outcomes. Together, these approaches highlight the potential of structure-guided and mechanistic therapeutic strategies to improve outcomes in cancers driven by dysregulation of AKT–mTOR signaling.

## Summary

The PI3K/AKT/mTOR signaling pathway is a central regulator of critical cellular functions, including growth, metabolism, survival, and protein synthesis. AKT, activated by PI3K signaling and its interaction with phosphoinositides such as PIP3 and PI3,4P2, orchestrates many of these processes through complex phosphorylation events mediated by PDK1 and mTORC2. mTORC1 and mTORC2 play distinct roles in cellular regulation, with mTORC1 driving protein synthesis and cell growth, while mTORC2 modulates AKT’s full activation through phosphorylation at Ser473. These pathways are tightly controlled by the PH domain of AKT, which ensures precise membrane targeting and activation, while also regulating its autoinhibition.

Dysregulation of this pathway, particularly through mutations or loss of regulatory proteins like PTEN, contributes significantly to disease progression, especially in cancer, by driving unchecked cell proliferation and survival. As a result, targeting the AKT/mTOR pathway has emerged as a promising strategy for therapeutic intervention. However, challenges remain in achieving selective and effective targeting due to the pathway’s complexity, isoform-specific signaling, and feedback mechanisms.

Emerging strategies, including combination therapies, offer hope for overcoming these challenges. A better understanding of AKT’s regulation and its interaction with mTOR complexes is essential for advancing these therapeutic approaches. By improving our knowledge of the PI3K/AKT/mTOR pathway, we can develop more precise and effective treatments, potentially transforming the management of diseases driven by its dysregulation, such as cancer. Future research should continue to refine these strategies, paving the way for personalized therapies with improved clinical outcomes.

## Data Availability

The data sharing is not applicable to the manuscript.

## Open Access

Open access for this article was enabled by the participation of Ohio State University in an all-inclusive Read & Publish agreement with Portland Press and the Biochemical Society under an agreement with the Big Ten Alliance.

## Abbreviations

ER: endoplasmic reticulum
GAP: GTPase-activating protein
INPP5: inositol polyphosphate-5-phosphatase
mTOR: mechanistic target of rapamycin
NF-kB: nuclear factor kappa-light-chain-enhancer of activated B cells
PDK1: 3-phosphoinositide-dependent protein kinase-1
PH: pleckstrin homology
PHLPP: phosphatase and tensin homolog-induced putative phosphatase
PI3K: phosphoinositide 3-kinase
PIKK: phosphoinositide 3-kinase-related kinase
PIP2: phosphatidylinositol-4,5-bisphosphate
PIP3: phosphatidylinositol-3,4,5-trisphosphate
PRAS40: Proline-rich Akt substrate of 40 kDa
PTM: posttranslational modification
Rheb: Ras homolog enriched in brain
RTK: receptor tyrosine kinase
SHIP: SH2-containing inositol phosphatase
TSC2: Tuberous Sclerosis Complex 2
UBL4A: ubiquitin-like protein 4A
